# Prediction of Lower Limb Kinetics and Kinematics during Walking by a Single IMU on the Lower Back Using Machine Learning

**DOI:** 10.3390/s20010130

**Published:** 2019-12-24

**Authors:** Hyerim Lim, Bumjoon Kim, Sukyung Park

**Affiliations:** Department of Mechanical Engineering, Korea Advanced Institute of Science and Technology (KAIST), Daejeon 34141, Korea; hyerimlim@kaist.ac.kr (H.L.); bumjoon.kim@kaist.ac.kr (B.K.)

**Keywords:** biomechanics, machine learning, walking, ground reaction forces, joint torques, spring mechanics, wearables

## Abstract

Recent studies have reported the application of artificial neural network (ANN) techniques on data of inertial measurement units (IMUs) to predict ground reaction forces (GRFs), which could serve as quantitative indicators of sports performance or rehabilitation. The number of IMUs and their measurement locations are often determined heuristically, and the rationale underlying the selection of these parameter values is not discussed. Using the dynamic relationship between the center of mass (CoM), the GRFs and joint kinetics, we propose the CoM as a single measurement location with which to predict the dynamic data of the lower limbs, using an ANN. Data from seven subjects walking on a treadmill at various speeds were collected from a single IMU worn near the sacrum. The data was segmented by step and numerically processed for integration. Six segment angles of the stance and swing leg, three joint torques, and two GRFs were estimated from the kinematics of the CoM measured from a single IMU sensor, with fair accuracy. These results indicate the importance of the CoM as a dynamic determinant of multi-segment kinetics during walking. The tradeoff between data quantity and wearable convenience can be solved by utilizing a machine learning algorithm based on the dynamic characteristics of human walking.

## 1. Introduction

Kinetic data of human motion, such as ground reaction forces (GRFs) and joint torques, serve as a quantitative indicator of sports performance or the effect of rehabilitation. GRFs and joint torques have served as indicators of injury risks and pain during running [1,2,3,4,5]. The progress of gait rehabilitation of hemiparetic patients has been monitored by the magnitude and the degree of asymmetry of the GRF [6,7,8]. The motion analysis used for these studies is performed in a laboratory with accurate motion trackers and force transducers, which often impose spatial and temporal constraints on subjects. 

With the development of sensor technology, the market for wearable motion monitoring systems has rapidly grown. Wearable motion monitoring products such as Galaxy, Garmin, Fitbit, and Apple watches monitor the overall rough motion information, such as the steps, cadence, distance, etc. That rough motion information, however, makes it difficult to estimate the kinetic information for the analysis of injury risks and rehabilitation. One of the common issues of current wearables is how to increase the quantity and quality of the measured data with minimal increase in the system complexity. In particular, obtaining force information using wearables is very challenging due to the size and weight of the force transducers. Instead of direct measurement, researchers have attempted to predict unmeasured GRF information from joint kinematics information. Oh et al. predicted 3D GRFs based on the whole body motion capture data, but the motion capture system still imposes spatial and temporal constraints on subjects [9]. To overcome the constraints of the motion capture system, Karatsidis et al. predicted GRFs using inertial measurement units (IMUs) on multiple limb segments, and fairly accurately estimated the 3D GRFs from walking speeds of 0.9 to 1.6 m/s [9,10].

The number of IMUs measuring whole body motion, however, greatly limits the applicability of the IMU based prediction method for wearable devices. To reduce the number of IMUs used for GRF prediction, recent studies used the artificial neural network (ANN) with a limited amount of measurement information. Using the ANN, researchers have utilized multi-IMU data located on the shank or foot of both legs to predict the 3D GRFs in walking and running [11,12]. Using a single IMU measurement taken at the sacrum, researchers predicted the vertical GRF [13] or the 3D GRF and used additional sensor information to detect gait events such as heel strike or toe-off [14]. The ANN could predict unmeasured force information from the IMUs, find the global optima of the loss function during backpropagation [15,16,17,18], generalize without overfitting [19,20], and select appropriate input types and size [10,21,22]. Selecting an appropriate type and size of the data plays a key role in improving training efficiency and test accuracy [21,22], and dimension reduction techniques or the employment of large datasets are often used for this purpose [10,23]. Oh et al. used self-organizing maps (SOM) and a generic algorithm-general regression neural network (GA-GRNN) to reduce the dimensionality of the input from 90 kinematic data points to 14, to predict 3D GRFs [10]. Johnson et al. collected kinematic and kinetic data from over 400 subjects, and used eight trajectories of the kinematic marker data, body parameters of mass and height, and gender information as inputs to the network [23]. Although a sufficiently large input data size may help with the search for appropriate input combinations, the search process often involves optimization and the use of heuristics. 

To resolve some issues of the ANN listed above, one may use knowledge of the dynamic characteristics of human walking. Whereas the multiple limbs used during walking are coordinated and actuated in a complicated manner by multiple muscles, the resultant motion of the body CoM and related forces are known to be describable by simple spring mechanics [24,25,26,27,28]. The experimental GRFs at various walking conditions are well emulated by the springy mechanics of the CoM [25,26], leading to the CoM motion being easily estimated by the GRF, and vice versa. By adding spring components and geometrical constraints to the spring-loaded inverted pendulum (SLIP) model, recent studies have shown the extended dynamic association of the CoM with the ankle joint torque and multiple joint kinetics [29], as well as swing leg kinetics [30]. These findings highlight the importance of the CoM as a dynamic determinant of multi-segment kinetics during gait. Noting that the ANN generates the output from the weighted sum of the inputs, the mechanical coupling between the CoM and the joint kinetics could be implemented in the ANN to efficiently and accurately predict the unmeasured joint kinetics.

In this study, using the dynamic relationship between the CoM, GRFs, and joint kinetics, we propose the CoM as a single measurement location which can be used to predict unmeasured dynamic data of the lower limbs using an ANN. Data from seven subjects walking on a treadmill at various speeds were collected from a single IMU worn near the sacrum. The data was segmented by step and numerically processed for integration. After training the ANN with the processed IMU data, lower limb kinematics and kinetics at various gait speeds were predicted and tested by the leave-one-subject-out (LOO) validation method.

## 2. Methods

**Figure 1:**
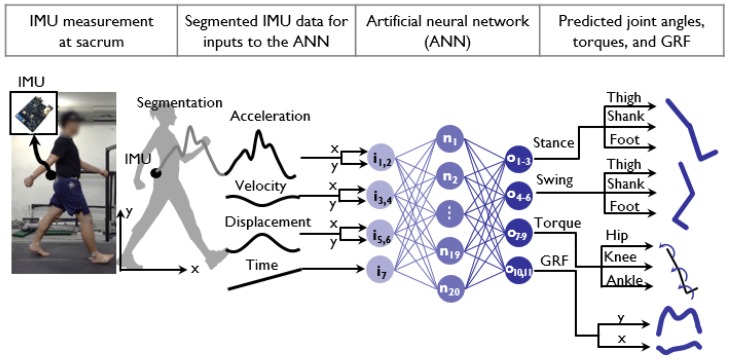
Schematics of the procedures of lower limb kinetics prediction from a single inertial measurement unit (IMU) using machine learning. Processed IMU signals, such as velocity, position and time, were fed into input nodes of the network. The network has seven input nodes followed by one hidden layer of 20 nodes and 11 output nodes. The output predictions are the angle of the thigh, shank, and foot of the stance and swing leg and the joint torques of the hip, knee, and ankle and the vertical and horizontal ground reaction forces.

We proposed a method for the prediction of six lower limb kinematics and five lower limb kinetics data points from a single IMU measurement using the ANN, based on the biomechanical characteristics of the CoM and the GRF during human walking (Figure 1). Mechanical coupling between the CoM, the GRF, and joint kinematics were used to approximate the GRF and joint motion as a weighted sum of the CoM, which is the basic input–output relationship of an ANN. We hypothesized that the CoM kinematics measured from a single IMU, used as an input node to the ANN, could predict the unmeasured GRF, joint kinematics and kinetics. The IMU measurement obtained from the sacrum was pre-processed to identify each step and to obtain position and velocity information. The processed IMU data were then fed into the input node of the ANN, which consists of a single hidden layer of 20 nodes with sigmoid activation and eleven output nodes for the segment angles of the foot, shank, and thigh of the stance and swing leg, the joint torques of the hip, knee, and ankle of the stance leg, and the horizontal and vertical GRF, respectively. The details of the experimental protocols, the signal processing of the IMU data, and the prediction algorithm are presented in Figure 1.

### 2.1. Finding the Location of a Single Measurement by the IMU

To examine which part of body motion should be measured to best estimate the lower limb joint kinetics, we used the dynamic characteristics of human walking. Since the outputs of the ANN are obtained from the weighted sum of the inputs passed through a nonlinear activation function, the equations of motion of the CoM during walking obtained from the recently published SLIP model [29] were approximated, to estimate the GRF and joint dynamics in terms of the weighted sum of the CoM. An approximation of the relationship between the CoM position and the state variables of the mass–spring model of human walking was formulated (Equation (1)). The GRFs and angles of multiple joints were formulated in terms of state variables (Equations (2) and (3)). Finally, the GRFs, ankle joint torque, and angles of the foot, shank, and thigh were formulated as the weighted sum of the CoM positions. These calculations led us to propose the CoM as the best measurement position for prediction of the unmeasured GRF and joint dynamics. A brief summary follows, the details of which can be found in the Appendix A.

**Figure 2:**
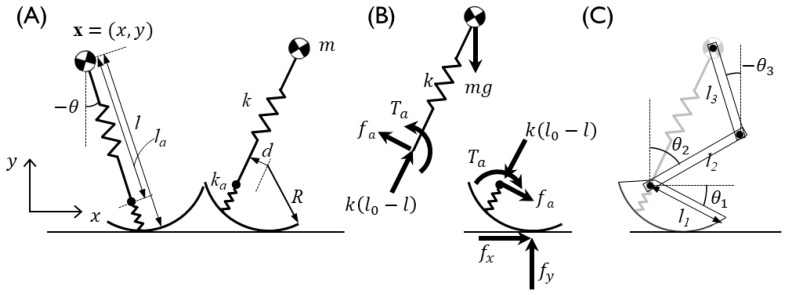
Compliant walking model with an off-centered, curvy foot combined with a springy foot-ankle segment [29]. (**A**) The model parameters and state variables of the model, and (**B**) the force and torque components. The position of the CoM in the sagittal plane; *x* and *y* are the positions of the horizontal and vertical axes, respectively, and the model state variables *l* and *θ* are the total spring length and angle, respectively, with a positive sign in the clockwise direction with respect to the vertical. The length and stiffness of the CoM–ankle are represented by *l_a_* and *k_a_*, respectively. The off-centered curvy foot parameters *d* and *R* are the offset from the ankle from the center of the foot and the radius of the curvy foot, respectively. The forces at the ground contact point are presented by *f_x_* and *f_y_* in the horizontal and vertical directions, respectively. The constraint force, *f_a_*, acts on the ankle in the vertical direction of the spring force, and the constraint ankle torque, *T_a_*, is generated to prevent foot rotation relative to the leg rotation, with a positive sign in the extension direction. With the kinematic constraint and the positions of the CoM and the ankle, (**C**) multibody segment angles are determined by inverse kinematics. Segment lengths and angles are represented by *l* and *θ*, respectively, with subscripts 1–3, and angles are positive in the clockwise direction.

The position of the CoM, x = (*x*, *y*), is expressed as model parameters of the SLIP, such as the leg angle *θ*, the leg length *l*, the radius of the curvy foot R, the offset of the ankle joint with respect to the foot d, and the ground contact position *c_hs_*, with the subscript *hs* denoting values at the onset of the stance phase (Figure 2), as follows:

By linearization, we can obtain the mapping equation from the CoM position **x** to the augmented state **z** = [*l*, *θ*, *lθ*]*^Τ^*, which includes the state parameters, *l* and *θ* of the SLIP model, and its nonlinear term *lθ* as follows:

(1) $$\begin{array}{l} z=W_{1}x+b_{1}\\ \mathrm{where}\;W_{1}=\left[\begin{array}{cc} 0 & 1\\ y_{hs}^{-1} & 0\\ 1 & 0 \end{array}\right],b_{1}=\left[\begin{array}{c} 0\\ \alpha y_{hs}^{-1}\\ \alpha \end{array}\right] \end{array}$$

where *α* = *Rθ_hs_* − *c_hs_* + *d.*

The relationship between the augmented state **z** and the augmented kinetics **f** = [*f_x_*, *f_y_*, *T_a_*]*^T^* such as GRF and ankle torque are obtained as follows:

(2) $$\begin{array}{c} f=W_{2}z+b_{2}\\ \mathrm{where}\;W_{2}=\left[\begin{array}{ccc} kdl_{0}{}^{-1} & k\left(l_{0}-R\right) & k\left(Rl_{0}{}^{-1}-1\right)\\ -k & kd & -kdl_{0}{}^{-1}\\ -kdc_{l} & kRl_{0}c_{l} & -kRc_{l} \end{array}\right],\;b_{2}=\left[\begin{array}{c} -kd\\ kl_{0}\\ kdl_{0}c_{l} \end{array}\right] \end{array}$$

The relationship between the augmented state **z** and the augmented segment angles **θ** = [*θ*_1_, *θ*_2_, *θ*_3_]*^T^* can be rewritten as follows:

(3) $$\begin{array}{l} \theta =W_{3}z+b_{3}\\ \mathrm{where}\;W_{3}=\left[\begin{array}{ccc} \left(1-c_{k}\right)l_{1}{}^{-1} & 1 & 0\\ \mp 0.25c_{l}c_{k}l_{0}l_{2}{}^{-2} & 0.5\left(c_{l}-c_{k}\right)l_{0}l_{2}{}^{-1} & 0.5c_{k}l_{2}{}^{-1}\\ \pm 0.25c_{l}c_{k}l_{0}l_{2}{}^{-2} & 0.5\left(c_{l}-c_{k}\right)l_{0}l_{2}{}^{-1} & 0.5c_{k}l_{2}{}^{-1} \end{array}\right],\\ b_{3}=\left[\begin{array}{c} -\left(1-c_{k}\right)l_{0}l_{1}{}^{-1}\\ \pm \left(1-0.125c_{l}\left(c_{l}-2c_{k}\right)l_{0}{}^{2}l_{2}{}^{-2}\right)\\ \mp \left(1-0.125c_{l}\left(c_{l}-2c_{k}\right)l_{0}{}^{2}l_{2}{}^{-2}\right) \end{array}\right] \end{array}$$

In summary, the kinematics and kinetics of the lower limb can be approximated as a weighted sum of augmented states **z** of the spring mechanics and the bias as follows:

(4) $$\left[\begin{array}{c} f\\ \theta \end{array}\right]=\left[\begin{array}{c} W_{2}W_{1}\\ W_{3}W_{1} \end{array}\right]x+\left[\begin{array}{c} W_{1}b_{1}+b_{2}\\ W_{3}b_{1}+b_{3} \end{array}\right]$$

From the approximated relationship between the CoM position **x** and the lower limb kinetics and kinematics [**f^T^**, **θ^T^**] as a form of weighted sum of x and bias, we tried to predict the unmeasured lower limb kinematics and kinetics using the sacrum motion profiles, the measurable approximation of the CoM. We may use the terms ‘CoM’ and ‘sacrum’ interchangeably throughout the manuscript (see also the Appendix A for the formulation of the lower limb kinematics and kinetics as an approximation of the weighted sum of the CoM states). 

### 2.2. Experimental Protocols and Data Collection

Seven young (25.0 ± 2.9 years) healthy male subjects with average heights and weights of 168.8 ± 7.5 cm and 72.1 ± 7.7 kg, respectively, participated in the data collection, after signing fully informed consent forms approved by KAIST Institutional Review Board on 22 January 2018 (IRB-18-023). The subjects participated in one-minute treadmill walking trials at three customized gait speeds, ranging from 1.0 m/s to 2.3 m/s. The test speeds were determined by each subject’s preferred gait speed, the midpoint between his preferred and maximum speeds, and a speed slower than the preferred speed by an amount equal to approximately 20% of the difference between the preferred and maximum speeds. The label ‘slow’, ‘moderate’, and ‘fast’ indicates slow speed, preferred speed, and fast speed, respectively. The speed of each subject is in Table 1. Three one-minute gait trials per test speed were randomly ordered. The kinematics of the lower back, which is assumed to be close to the CoM, were measured by an IMU (EBIMU-9DOFV4) with an acceleration sensitivity of ±16 g, and resolution of 0.001 g. The width of the IMU was 15.6 mm, and the length was 18.6 mm. The IMU data were collected at a sampling frequency of 100 Hz, and used the universal asynchronous receiver/transmitter (UART) protocol for communication with a baud rate of 921,600 bps. The IMU was aligned with the subject’s heading direction to set the local frame of the IMU to the body frame. The 2D acceleration data from the IMU in the sagittal plane were used for the analysis. The lower limb kinematics and ground reaction forces under each foot were measured by an optical motion capture system (Motion Analysis Hawk^®^) and force plates (Bertec, FP 6012^®^), respectively. For the segment angle calculation, 12 markers were located at the toe, heel, ankle, knee, and anterior and posterior superior iliac spine (ASI and PSI) of both legs. The midpoint of the PSIs was used as the reference position for the IMU. Motion data and force data were collected at the sampling frequency of 100 Hz and 200 Hz, respectively, and filtered with a fifth-order low-pass Butterworth filter with a cutoff frequency of 10 Hz. The joint torque was calculated by the inverse dynamics of the seven-segmental rigid body model, which includes the hip, knee, and ankle. The mass distribution of the rigid body were based on the anatomical reports [31].

### 2.3. Preprocessing of IMU Data for ANN Input

**Figure 3:**
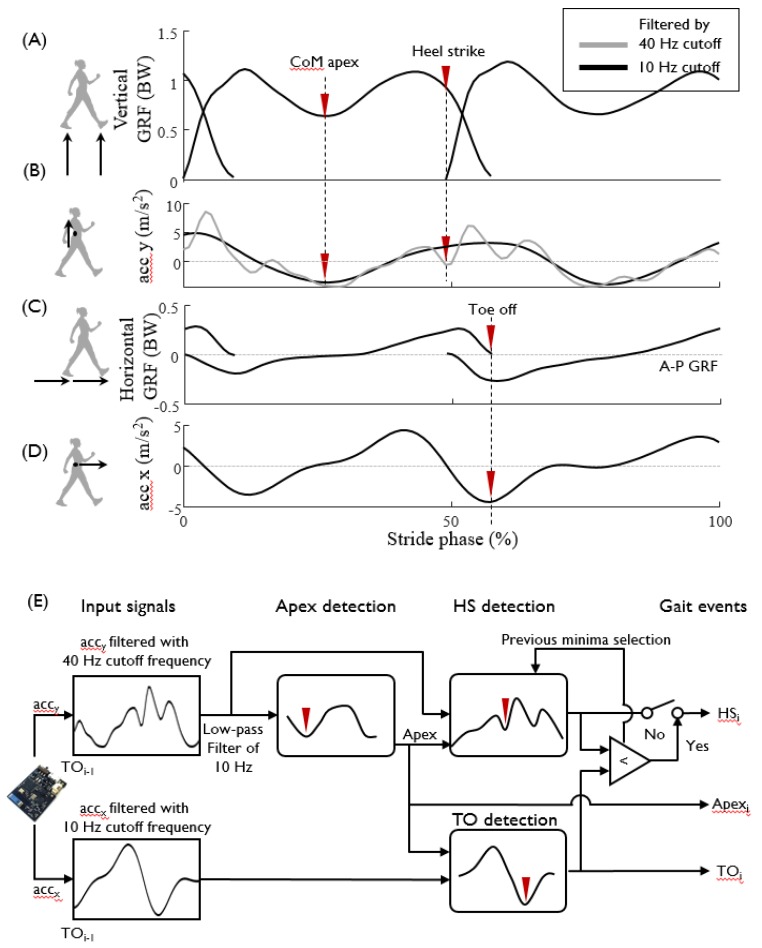
Schematics of the gait event detection and IMU data segmentation algorithm. To detect the gait events of heel strike (HS) and toe off (TO), the acceleration measurement in the (**B**) vertical and (**D**) anteroposterior (AP) directions is compared with the (**A**) vertical and (**C**) eranteroposterior GRF measurement. Timings of specific gait events, such as the CoM apex, HS, and TO, are marked with reversed triangle. Data filtered by 10 Hz and 40 Hz cutoff frequencies are presented by black and gray solid lines, respectively. (**E**) Gait event detection algorithm. From the local and global minimum points of the vertical and A–P accelerations (see Methods section), the apex, HS and TO events are detected.

The IMU measurement data that contain the double support phase must be segmented into one stance phase per leg before feeding into the ANN for GRF prediction. The stance phase is defined based on the GRF as the duration from the onset of heel strike (HS) to toe off (TO) (Figure 3A,C). To identify the characteristic features of the IMU data which indicate the gait events of HS and TO, the IMU acceleration and GRF profiles were aligned over time, and three characteristic features were chosen: the CoM apex, HS, and TO (Figure 3). The filter frequency of IMU acceleration was selected heuristically to best extract the characteristic features, and was chosen to be 40 Hz for identifying HS and 10 Hz for identifying TO. The CoM apex is the point at which the height of CoM is maximal in sagittal plane around the mid-stance phase, and the corresponding vertical GRF has a local minimum (Figure 3B). The CoM apex was identified as the local minimum of the vertical IMU acceleration data filtered with a cutoff frequency of 10 Hz (Figure 3B). The HS was identified as the first local minimum of the vertical acceleration filtered by a 40 Hz cutoff frequency, following the positive local maximum after CoM apex (Figure 3B). The TO was identified as the first local minimum of the horizontal acceleration data filtered by a 10 Hz cutoff frequency after CoM apex (Figure 3D). The detailed process of IMU data segmentation is shown in Figure 3E. The precedence of HS over TO was checked for validation. When HS estimation follows the TO, the HS was re-identified as the preceding local minimum of vertical acceleration compared with the one originally found. After completing segmentation of the full stance, the consecutive segmentation process was performed by identifying the subsequent CoM apex based on the acceleration profiles from the TO event, the last event of the previous step. 

Another step of preprocessing the IMU data is to calculate the velocity and position of the CoM, i.e., the sacrum, from the acceleration, which includes drift removal and identification of the integral constant. To eliminate the integration drift, steady walking and random error were assumed. Then, the drift was removed linearly with time over the duration from one apex to the following apex. The drift-removed velocity of the CoM $\hat{v}(t)$ could be obtained as follows:

(5) $$\hat{v}(t)=v(t)-\left(v(T)-v(0)\right)\cdot \frac{t}{T}$$

where **v** (*t*) is the time integration of the acceleration data for time *t* over the duration of the stance phase *T*. To satisfy the steady walking assumption, the mean velocity change was set to zero so that position drift due to the random error was simultaneously removed.

The velocity and the position offsets were estimated in a heuristic manner to obtain the velocity and the position from the acceleration integration. From the observed proportionality of the gait speed v with the average magnitude of the acceleration, *A*, and the gait frequency, *f*, (0.79 and 0.73 Pearson correlation coefficients, respectively), the gait speed offset *v*_0_ was estimated as follows:

(6) $$v_{0}=\frac{aA+bf+c}{2}\cdot \sqrt{h\cdot g}$$

where *a*, *b*, and *c* are the regression coefficients obtained in a least square manner. To minimize the size effect of subjects, we normalized the speed with $\sqrt{h\cdot g}$, where *h* and *g* are the height of the subject and gravitational acceleration, respectively. The position of the sacrum was obtained by integrating the compensated velocity with the position offset, defined as the subject’s leg length. All data processing was conducted by MATLAB R2018a (Mathworks, Inc., Natick, MA, USA).

### 2.4. Structure of the ANN and Its Training and Test Procedures

The GRFs and segment angles of the lower limbs were approximated using the weighted sum of CoM position (Equation (4)), so we hypothesized that a simple ANN that generates its output from the weighted sum of inputs could be a good candidate for a prediction network. We used a fully connected feed-forward ANN with one input layer, one hidden layer and one output layer. The seven input nodes consist of a 7 × 1 column vector consisting of a time *t*, and the corresponding CoM kinematics *(t*, *x*, *y*, *v_x_*, *v_y_*, *a_x_*, *a_y_*), where *x*, *y* are horizontal and vertical position, respectively, and v and a are velocity and acceleration measured at a specific time, such as *t* = *t*_0_. The position of the CoM was reset to be zero at every HS, and we defined ‘displacement’ as the reset position. The eleven output nodes constitute an 11 × 1 column vector consisting of the GRFs and the joint kinetics and kinematics corresponding to *t* = *t*_0_, such as (*GRF*_x_, *GRF*_y_, *T*_ank_, *T*_knee_, *T*_hip_, *θ*_foot,stance_, *θ*_shank,stance_, *θ*_thigh,stance_, *θ*_foot,swing_, *θ*_shank,swing_, *θ*_thigh,swing_), where *T*, and *θ* are joint torque and segment angle, respectively, with subscripts showing the names of the lower limb segment. All kinematic data of the sacrum, such as the acceleration, velocity and displacement, were normalized by its maximum norm such that the values ranged from 0 to 1. The hidden layer has 20 nodes, and node weights are iteratively updated to best match the data. The sigmoid and linear activation transfer functions were used in the hidden and output layers. The loss function of the network was set as the mean square error of the predicted angles of both the stance and swing legs, the joint torque of the stance leg and the GRF with respect to the observed data. The network employed an Adam optimizer for the back-propagation process. To match the size of the training dataset collected at various gait speeds of many subjects, gait data of one stance phase were interpolated into 200 points. As a quantitative measure of prediction performance, we used the normalized root mean square error (NRMSE), which was normalized by the difference between the maximum and minimum values of the outputs of each subject at each speed. The NRMSE value of each subject represents the error of a total of 30 trials at one speed for one output. The neural network programming was conducted using PyTorch 0.4.1 [32].

To test the proposed prediction methods, we used LOO cross validation. A total of 540 stance phase trials obtained from six subjects was used as the training dataset, and the results were validated using data from the remaining subject. Validation was performed for each of seven subjects. To evaluate which kinematic information contributed most to the prediction accuracy, various combinations of kinematic inputs, such as CoM kinematics with or without position, velocity, and acceleration, were used as the inputs to the ANN. To examine whether the walking speed at which the training datasets were collected affected the prediction accuracy of the GRFs at other speeds, the data collected at various walking speeds were used as training datasets of the ANN.

## 3. Results

From the single IMU measurement at the lower back, the practical approximate of the CoM, eleven joint kinetics data points were predicted by the ANN over walking speeds ranging from 1.0 to 2.3 m/s. The predicted joint data are the thigh, shank and foot angles of the swing and stance leg, the hip, knee, and ankle joint torques of the stance leg, and the vertical and A–P ground reaction forces. 

**Figure 4:**
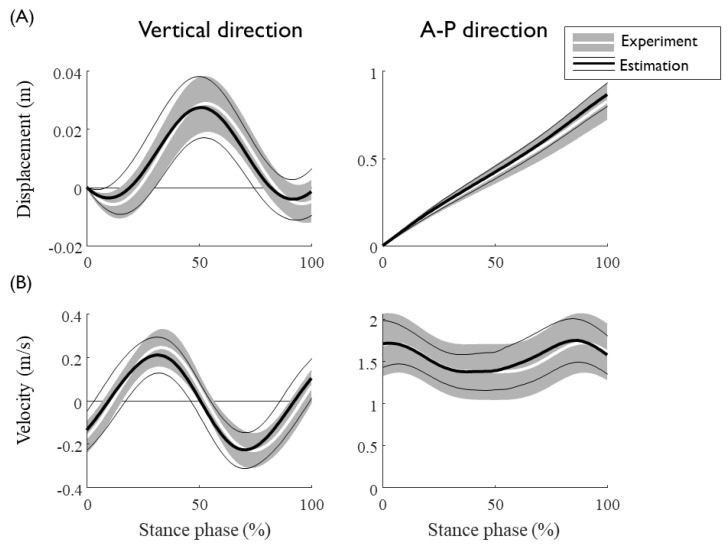
The average trajectories of (**A**) displacement and (**B**) speed of the CoM over 90 trials of seven subjects at gait speeds ranging from 1.0 to 2.3 m/s. Vertical and horizontal components are presented in the left and right columns, respectively. The experimental mean and standard deviation are depicted as gray shaded and white solid lines, respectively, whereas the estimated values are depicted as thick and thin black solid lines, respectively.

Gait event detection from the IMU data showed a reasonable match with those defined from the GRF, with average detection errors of the HS and TO of 0.025 s and 0.014 s, respectively (Table 2). The detection algorithm showed a more accurate performance for TO than that for HS, with the accuracy increasing with the gait speed. With drift removal and the estimation of the integral constant of the IMU measured acceleration, the velocity and displacement trajectories of the CoM were obtained by integrating the IMU data based on the detected gait events (Figure 4). The calculated sacrum speed and the displacement segmented by the stance phase matched well with the data obtained from optical markers, with NRMSEs of approximately 25% and 12%, respectively (Table 3). No significant tendencies were observed for the estimation errors of the sacrum kinematics as a function of the gait speed.

**Figure 5:**
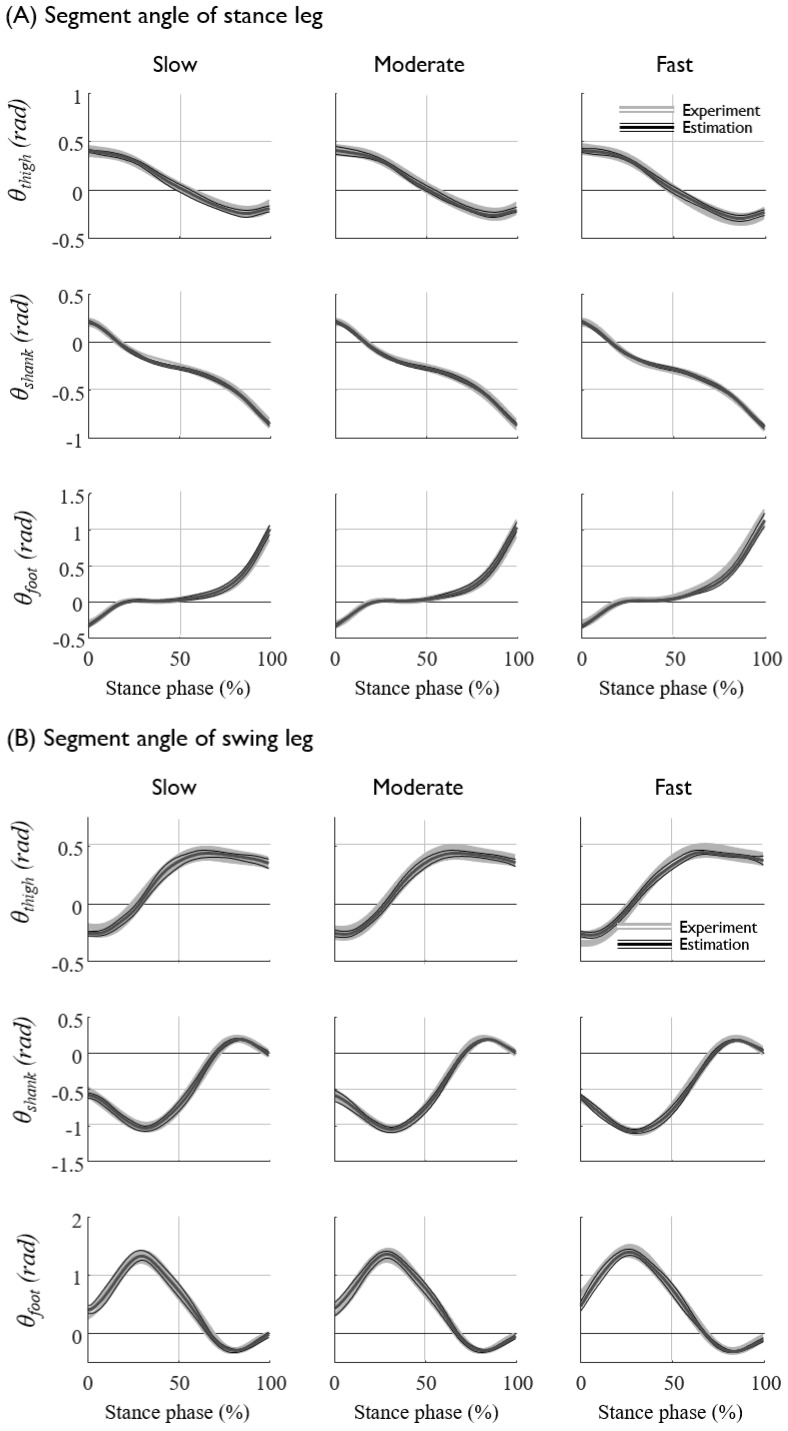

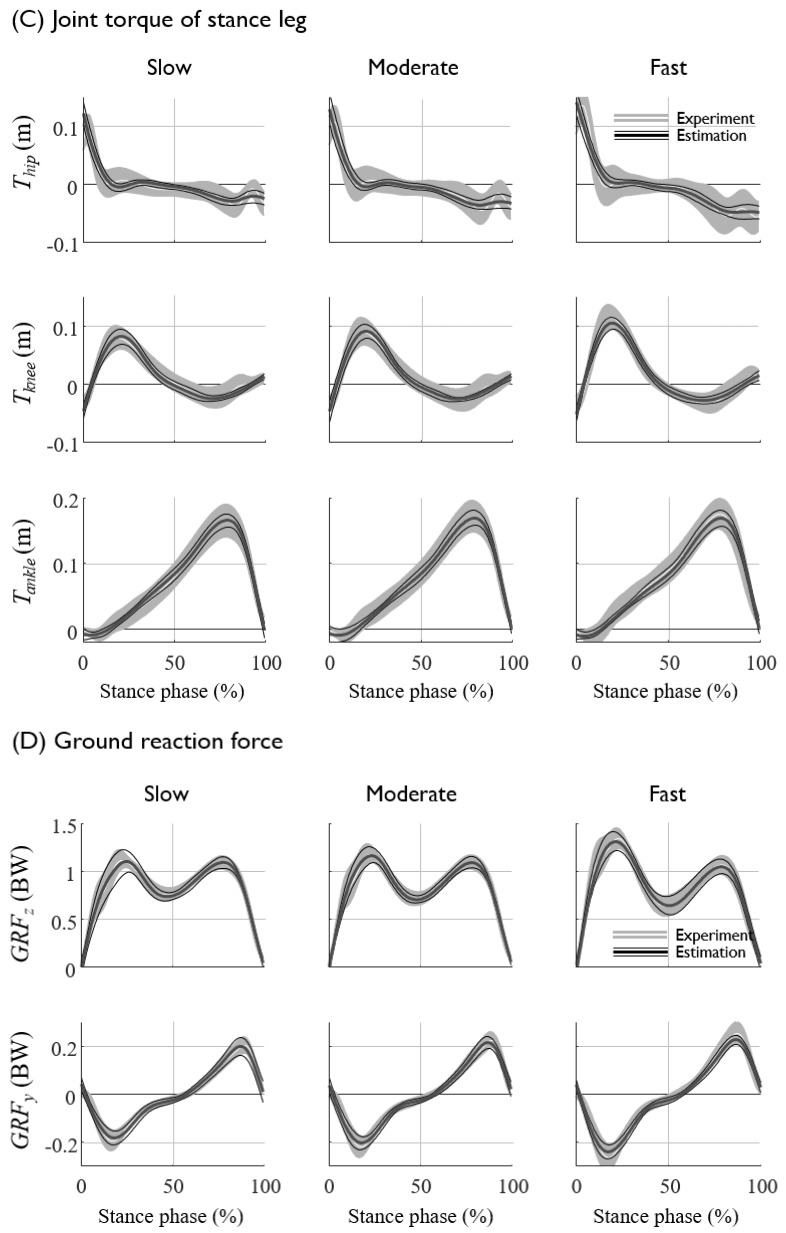
The prediction results and experimental data of (**A**) the segment angles of the stance leg, (**B**) swing leg, (**C**) the joint torques of the stance leg, and (**D**) the ground reaction forces, averaged for all seven subjects. The mean and standard deviations of the experimental data are depicted as white solid lines with a gray shadow, and those of the prediction results are shown as thick and thin black solid lines, respectively.

**Figure 6:**
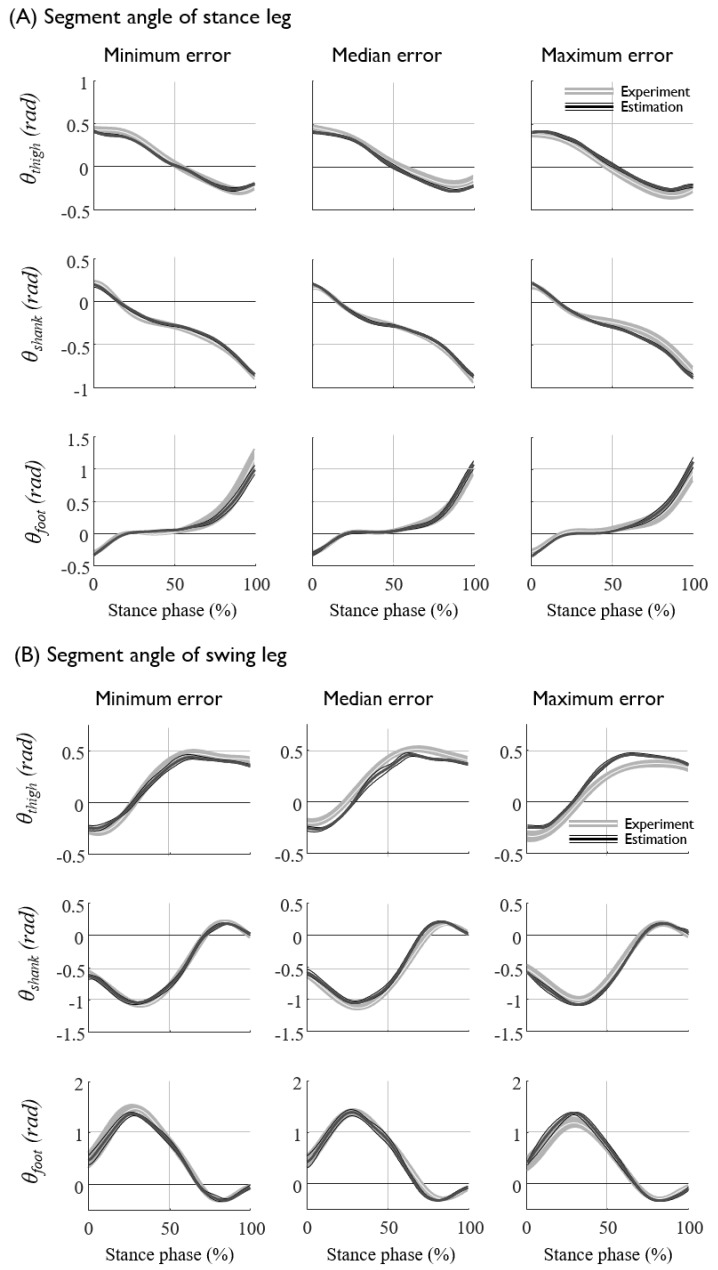

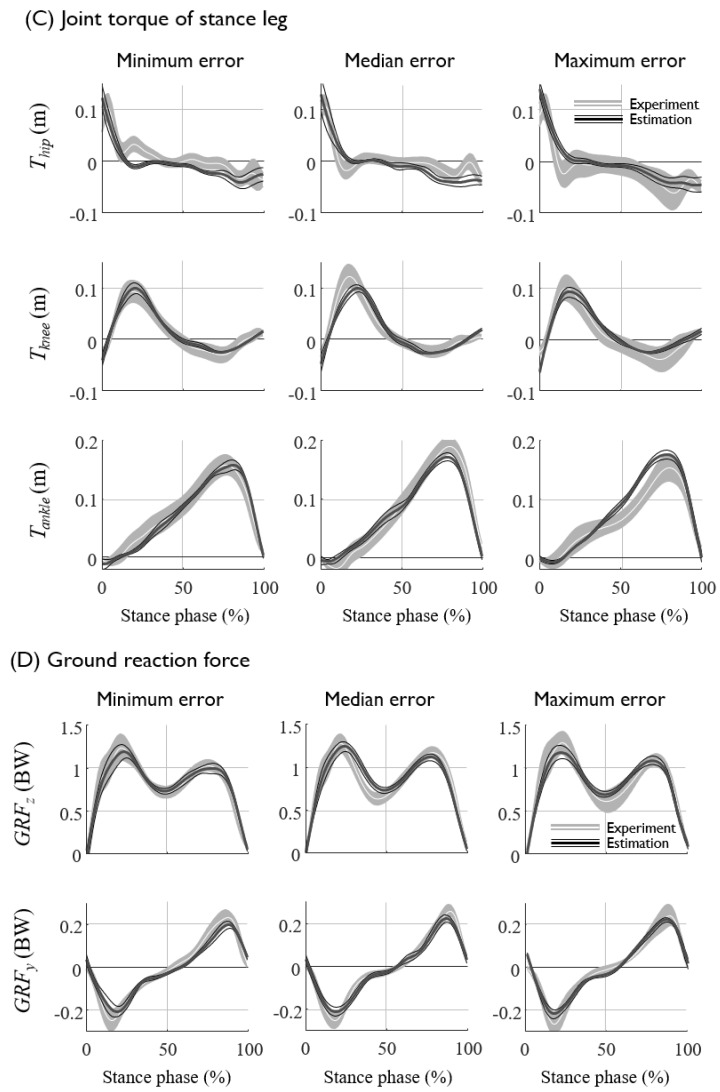
The prediction results and experimental data of (**A**) the segment angles of the stance leg, (**B**) swing leg, (**C**) the joint torques of the stance leg, and (**D**) the ground reaction forces. The mean and standard deviations of the experimental data are depicted as a white solid line with a gray shadow, and those of the prediction results are shown as thick and thin black solid lines, respectively. The data correspond to three subjects with different levels of estimation errors of the minimum (NRMSE: 6.15 ± 2.52%), median (NRMSE: 7.11 ± 2.70%), and maximum errors (NRMSE: 8.21 ± 2.81%), shown from left to right. The graph shows the average trajectories of 90 trials per subject collected at various (slow, moderate, and fast) gait speeds.

**Figure 7:**
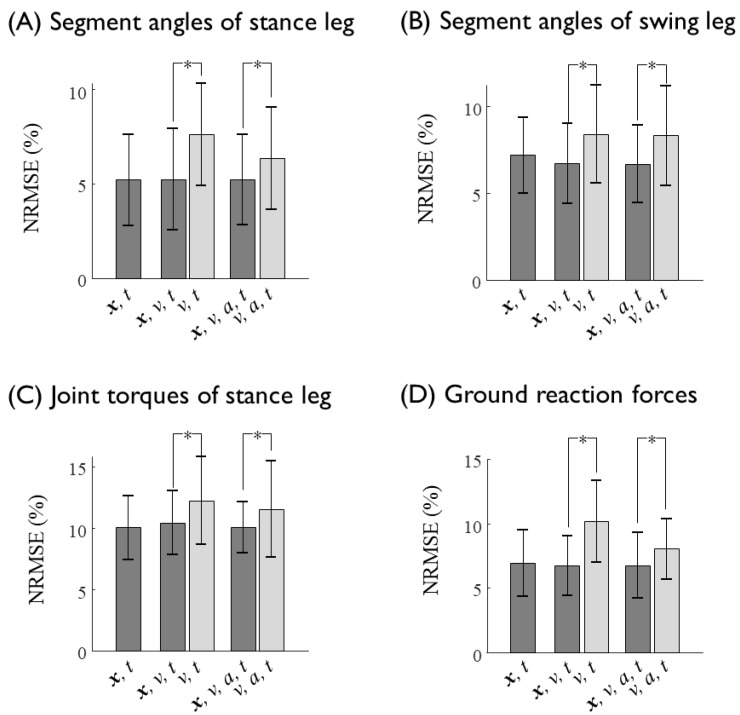
Normalized root mean square errors (NRMSEs) of the estimation of (**A**) the segment angles of the stance leg, (**B**) the segment angles of the swing leg, (**C**) the joint torques of the stance leg, and (**D**) the ground reaction forces as a function of the input variables fed into the neural network. Prediction errors in response to the ANN input data with and without displacement (*x*) of the sacrum are shown as black and gray bar graphs, respectively. Asterisk shows statistical significance (*p* < 0.05).

The prediction results (NRMSE and $R^{2}$) of each subjects are presented in Table A1 and Table A2 in the Appendix A. The lower-limb joint kinematics and kinetics at various gait speeds for all seven subjects were fairly well predicted from the kinematics of the sacrum, such as the displacement, velocity, and acceleration, with an average NRMSE of approximately 7% (Figure 5 and Figure 6, Table 4). The segment angles of the stance and swing leg showed a reasonably good match between the prediction and experimental data, even for the worst case test set (Figure 6A,B, Table 5), whereas the joint torques and GRFs showed larger error, especially for the estimation of the high-frequency component of the hip joint torques (Figure 6C,D, Table 5). The average prediction errors also showed a similar trend in the prediction accuracy (Figure 5, Table 4). As long as the displacement information of the sacrum is provided, the velocity and acceleration input to the ANN do not significantly improve the prediction accuracy, whereas the omission of the displacement input significantly (*p* < 0.05) increases the prediction errors of the segment angles, torques and GRFs up to 3% (Figure 7, Table 6). These results imply that the position of the sacrum contributes most to the predictions of the lower-limb joint kinetics. 

**Figure 8:**
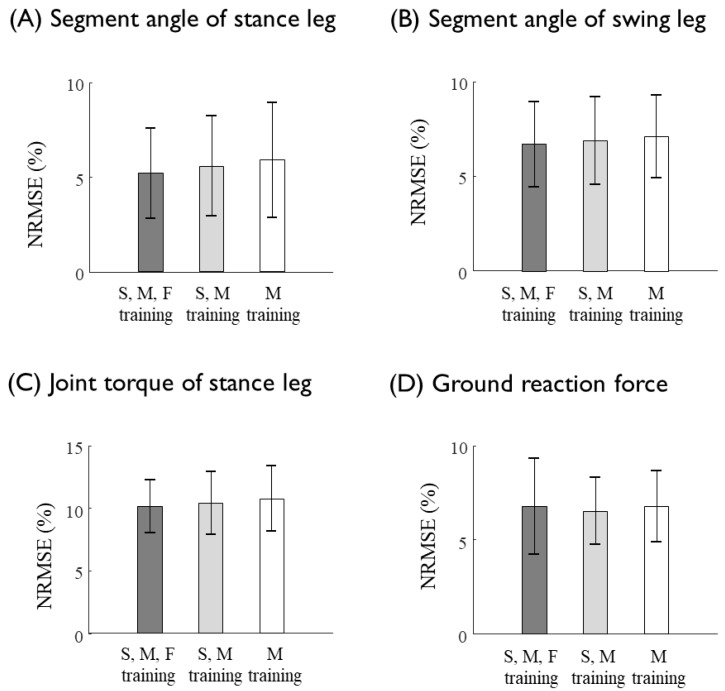
Prediction errors (NRMSEs) of (**A**) the segment angles of the stance leg, (**B**) the segment angles of the swing leg, (**C**) the joint torques of the stance leg, and (**D**) the GRFs at various (slow, moderate, and fast) gait speeds. The ANN was trained with data collected at slow, moderate and fast speeds (dark gray bars), slow and moderate speeds (light gray bars), and moderate speed only (white bar). There was no statistically significant difference.

**Figure 9:**
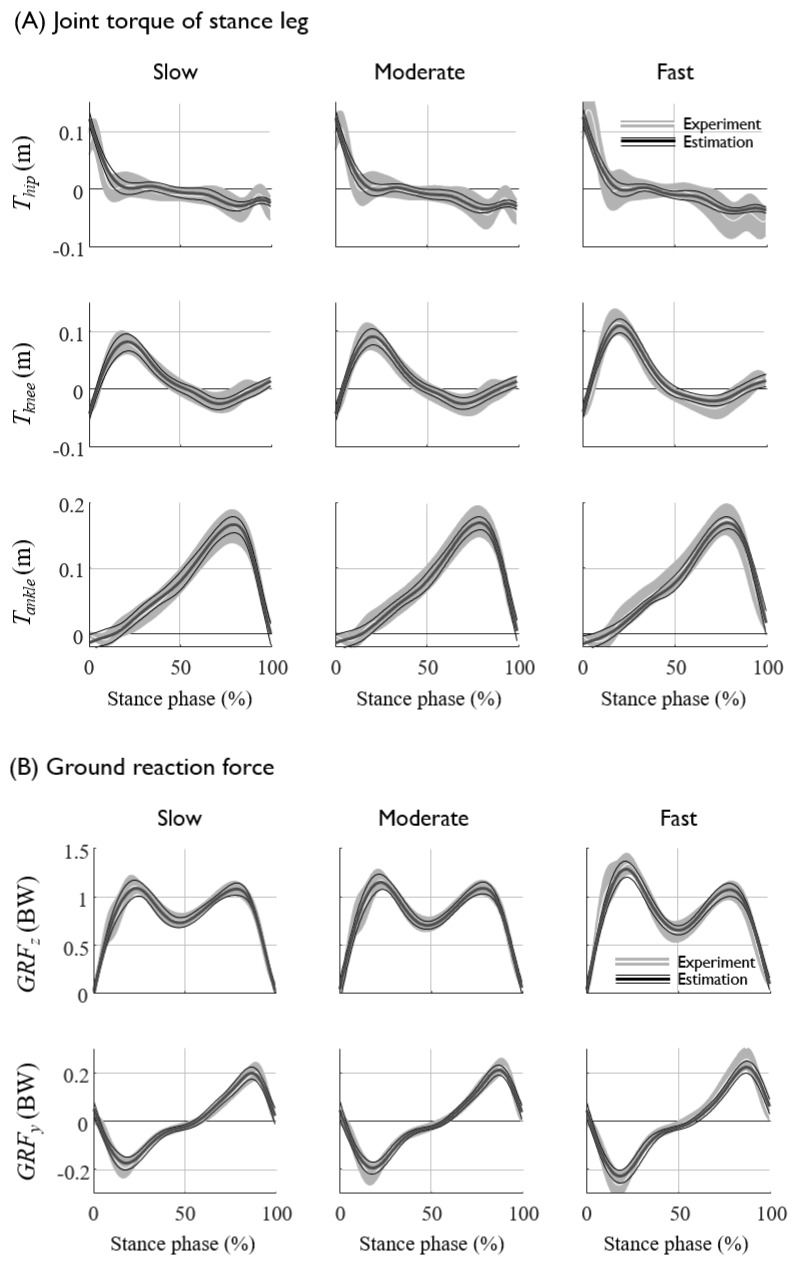
The experimental data and the prediction data of the (**A**) joint torques and (**B**) GRFs at various gait speed obtained from the ANN trained by trials of moderate speed only. The average and standard deviations of the experimental data are represented by a white line and gray shaded area, respectively, and those of the estimation are represented by thick and thin black lines, respectively.

The ANN predicted the kinematics and kinetics of the lower limb at various gait speeds that were not used for the training datasets. The joint kinematics and kinetics are known to vary as a function of the walking speed. To examine the effect of the walking speed at which the training data sets are collected to the prediction accuracy, the ANN was trained by the data collected at three different speed conditions: at moderate speed only, at slow and moderate speeds, and at slow, moderate and fast speeds (Figure 8). The results showed that the inclusion of the data of a specific gait speed, which are to be predicted through the ANN, would slightly but not significantly increase the prediction performance. More importantly, the sacrum kinematics measured at the moderate speed could predict the eleven unmeasured joint kinetics at various walking speeds, which were not included in the training of the ANN (Figure 8). The prediction results of the joint torques and GRFs at the various gait speeds obtained from the ANN trained by trials of moderate speed showed a reasonable match with the data (Figure 9, Table 7).

## 4. Discussion

Biomechanical knowledge about walking dynamics was used to design an ANN to predict unmeasured motion data from a single IMU measurement. Equations related to CoM dynamics were reformulated to approximate the GRF and lower limb joint dynamics as the weighted sum of the CoM, the basic input–output relationship of the ANN. Based on this approximation, the ANN was designed to have input nodes receiving the kinematics of the CoM, with the layers being fully connected. Using this ANN, eleven lower limb joint dynamics and the GRF data at various walking speeds were predicted from a single and only IMU measurement near the sacrum. The predicted segment angles, joint torques, and GRF data showed a reasonably good match with the experimental data despite the very simple network structure (Figure 1) with a relatively small number of training data sets (Figure 5, Figure 6, Figure 7 and Figure 8). Although trained with the CoM data measured at a specific gait speed, the ANN could predict multiple joint kinetics at various gait speeds that have not been used for network training. These results imply that the CoM is important as a dynamic determinant of multi-segment kinetics during walking, and that the tradeoff between data quantity and wearable convenience can be solved by utilizing a machine learning algorithm based on the dynamic characteristics of human walking. 

Based on a biomechanical analysis using 2D spring mechanics, we showed that the 2D acceleration data of the CoM could predict the GRFs and joint dynamics in 2D fairly well. Due to the point mass assumption of the CoM, no rotational motion of the CoM was assumed, and no gyroscope data were used for prediction. We may improve the quality of the prediction heuristically with the full usage of IMU sensors, such as 3D accelerations and 3D angular rates from the gyroscope. We do not, however, have sound explanations as to why the addition of 3D data would help in obtaining better prediction. We are investigating whether the 3D GRF could be well formulated using 3D CoM kinematics as a form of simple mechanics, as was the case for 2D. Despite the lack of quantitative formulation, there is considerable experimental evidence to show that full usage of the IMU data could predict the unmeasured GRFs, metabolic cost, and joint angles [9,33,34]. Three dimensional acceleration and angular rate measured from the 17 IMUs attached to the whole body have been used to successfully predict 3D GRF data [9]. Similar to our study, the 2D joint angles of lower limbs have been estimated from IMU measurement at the foot, shank, and thigh [34]. The 3D acceleration and gyroscope data were processed using general regression neural networks and the authors found that foot acceleration was sufficient to predict the other joint angles. Despite the larger number of sensors, the prediction errors were slightly larger (4~9 degrees) than ours (2~3 degrees), implying that the CoM kinematics are closely related to lower limb kinematics. The predicted quantities were not limited to the GRF or joint kinematics. Metabolic cost is one of the key determinants for human motion [35,36,37,38]. Measurements of energy expenditure, however, are limited to indoor settings, and the measurement procedures are often tedious. Researchers attempted to estimate the metabolic cost during walking from IMU data measured at the hip joint using Bayesian linear regression [33]. With the support of quantitative biomechanical analysis of the target motion to be predicted, the IMU data could be used more effectively and efficiently. There are studies that use machine learning technology to predict human motion, independent of gait biomechanics, but often with the cost of increased measurement complexity of the prediction errors. Oh et al. successfully predicted the joint torques and GRFs of human walking using a general regression neural network (GRNN), with the cost of increased measurement of the whole body joint kinematics using an optical marker system [10], which is therefore limited for on-field applications. The errors of the vertical and A–P GRF were comparable to ours (Table 8). Recently, without a great loss of prediction accuracy, the number of IMUs were reduced to two, attached at each shank of the subject [11]. To complement the reduced sensor information, a feed-forward neural network was used to produce the best estimate of the GRFs. In this recent study, however, the training datasets included part of the data from the test subject [11], which may have decreased the prediction accuracy when LOO validation was performed. Because the GRF pattern could differ between subjects [39,40], we used a conservative validation method, by excluding the test subjects’ data from the training dataset, and following the LOO validation method to guarantee generalization.

Machine learning techniques have been used to estimate joint angles and joint torques (Table 9) [34]. Luu et al. used GRNN and inverse fast Fourier transforms [41]. The predicted joint angles of lower limbs showed relatively small errors, in the range of 3~5 degrees, compared to others, but the test data were included in the training data set, which would produce less-accurate predictions when LOO validation is performed. Joint torque predictions have been performed using joint angle data [42] as well as surface electromyography (sEMG) [43]. The errors of the joint torque predictions were larger than those of GRFs and/or joint angles, as was observed in our study (Table 10). Ardestani et al. used 14 sEMG data and showed relatively high accuracy in joint torque prediction [43], but, again, the inclusion of test data in the training dataset of the four subjects may contribute to a misleadingly high prediction accuracy. Compared to those of related works, we predicted more data at various walking speeds with fewer measurements from the complementary combination of the biomechanics and the ANN.

The proposed prediction method using a combination of biomechanics and machine learning could be used to resolve the tradeoff between data richness and measurement convenience inherent in wearable technologies. The rehabilitation success of stroke patients can be assessed using the A–P direction of the GRF of the nonparametric leg [6,7,8], which would be challenging to measure if patients have to regularly visit a motion analysis laboratory and undergo almost over an hour of data collection procedures. Motion monitoring of those patients using simple wearables during their clinical visits or even at home in daily life would greatly increase patients’ convenience, if the data from wearables could produce data of quality and quantity comparable to those obtained in the lab. A recent study demonstrated successful long-term monitoring of the range of motion (ROM) of the shoulders of elderly people using the IMU, which showed a fairly good match with data from optical motion capture systems [44]. Similarly, onsite monitoring of lower limb joint loading could be estimated from a single IMU sensor for patients with rheumatoid arthritis or anterior cruciate ligament recovery. As a quantitative comparison, the asymmetry of the GRFs of mild hemiparesis patients seemed to be approximated as 50 N [7]. Kesar et al. proposed minimal detectable changes of the kinematic and kinetic indicators of rehabilitation assessment of post-stroke patients to be about 3–10 degrees and 20–33 N, respectively [45]. In our study, the average prediction errors of segment angles are about (3.1 degree) thigh, (2.2 degree) shank and (3.4 degree) foot, respectively. The approximate errors of vertical and AP GRF are 58 N and 23 N, and those of hip, knee, ankle joint torques are 16.7 Nm, 11.4 Nm, and 15.3 Nm, respectively. Considering the observed changes of GRFs and joint kinematics of stroke patients, the proposed method could serve well for kinematics monitoring, whereas the GRF monitoring seemed to be feasible for A–P GRF but need improvement for vertical GRF. In addition to the rehabilitation research, the number of studies monitoring sports activity is rapidly increasing. Wearable monitoring systems are widely used for monitoring the effect of posture or loading conditions on kinetics [46]. Data from sacrum-mounted IMUs were used to analyze the effect of load carriage and fatigue on jumping motions. The change of kinematics due to fatigue was similar to that under load conditions. IMUs on the lower body were used to estimate joint kinematics and GRF during running [47,48]. Combining single IMU data from the sacrum with a convolutional neural network, Johnson et al. estimated 3D GRF with the error of 4–9% during running and side-stepping [14]. A leg-mounted IMU combined with a feed forward neural network predicted knee joint forces with an error rate of 17% during running, changing direction, and jumping [49]. Issues to be addressed for sports wearables include reducing the number of sensors and enhancing prediction accuracy. The difference in kinetics with different foot strike patterns in running is about 10%–20% of joint torque and 30% of GRF. [50]. A recent study showed that a magnitude of about 30% of GRF in response to the breaking force could serve as an indicator for injury risk [51]. By assuming reference force and length to be the average weight and height of our test subjects, the observed kinetic differences range approximately 200 N and 18 Nm, respectively. Considering the error range of our study, the proposed prediction method can be applicable for monitoring running.

The limitations of our study, and technical issues to be resolved, lie in the main components of the prediction method, such as the biomechanical model, the processing of the IMU data, and the design of the ANN. First, the proposed prediction method utilized a spring-based biomechanical model of CoM dynamics during steady gait trials in 2D. Pathological gaits, however, often show increased asymmetry in the medial–lateral (M–L) as well as the A–P direction [52]. Thus, further research is required into applying the proposed method to the monitoring of unsteady or abnormal walking in 3D. Biomechanical modeling of human walking with a passive spring and asymmetric foot could well emulate the human-like ankle joint torque [29], implying that the CoM and ankle joint torque are mechanically coupled. The biomechanical model of the point mass CoM, however, could not represent the activation of hip joint torques, so there is no mechanical and kinematical correlation between the CoM motion and the hip joint torques. Since the ANN used for joint torque prediction was designed based on the biomechanics of the CoM, the larger prediction errors in hip joint torque presented in Figure 9A could be attributed to the limited representability of the hip joint torque by the CoM. Secondly, one of technical challenges of handling IMU data is the determination of appropriate and accurate segmentation. For long-term IMU data from motion monitoring, the very first step to perform in signal processing is to extract meaningful data from a whole data collection. A recent study used GPS information to specify the data segment of the action of interest out of approximately two weeks of IMU data in daily life [24]. To expand the proposed prediction method to gait monitoring during daily life, an appropriate activity recognition and extraction process must be undertaken. Once the data were roughly segmented for a specific action, such as walking in the hallway, further segmentation was carried out based on specific gait events, such as HS or TO. IMU data gathered near a ground contact such as the shank or foot had more accurate event detection results than when the IMU was mounted near the sacrum or lower back [53,54] possibly due to the distal measurement of the indicating signal around the foot [54,55,56]. Similar to previous studies, the identification of gait events and segmentation of the data were performed in a heuristic manner, but in a simpler way than those presented in other studies, such as those using wavelet transforms [57]. For example, the filtering frequency of the IMU data was selected heuristically by choosing the one that did not smooth the indicating signal of the gait event, while not including unnecessary peaks. The proposed event detection method produced errors comparable to those of previous studies, ranging from approximately 0.01–0.03 seconds [53,54]. The detection errors were reduced with gait speed due to the highlighted singular motion of the sacrum at faster speeds [28]. The gait event detection mostly follows the real gait event due to the delayed sacrum acceleration profile compared to that of the true CoM acceleration, which is obtained from the GRF, and resulted in shifted estimation of sacrum trajectory. Likewise, although small in magnitude, event detection errors could greatly affect the prediction results when erroneously segmented IMU data are used for training an ANN. An ANN is highly dependent on its input information, so poorly-estimated sacrum kinematics cause a deterioration in prediction accuracy. Thus, the prediction performance is sensitively dependent on the signal quality of the IMU and the following signal processing. Considering that supervised learning is performed between the segmented IMU data and the segmented GRF, erroneously segmented IMU data distort the coupling relationship between the CoM and the GRF, and the network will be trained inaccurately and inefficiently. Further, there was a relatively large error (~25%) in the velocity estimation (Figure 4). The integration of the acceleration had drift removal and offset compensation issues, which were resolved in a heuristic manner. Thorough examination of the best estimation of the gait speed was not performed in this study, so the error rate could potentially be reduced by employing other estimation algorithms or machine learning techniques. The effects of erroneous velocity estimation on the prediction results, however, are reduced when the velocity is fed into the ANN after normalization. In addition, the validation conducted depends on the location of IMU. Since the trunk has rotational motion with respect to the vertical axis, the location of IMU in the horizontal axis can affect the prediction performance. Thus, the sensitivity analysis about the location of IMU should be performed for validation of the reliable wearable motion monitoring system. Lastly, the proposed ANN has much room for improvement of the prediction accuracy. The proposed ANN is a very simple network, designed using a simplified mathematical approximation between the GRFs and the CoM. A more realistic approximation could be achieved by adding complexity to the network, such as additional input nodes or hidden layers, or a change of activation function. A relatively small number of training datasets, and reasonably good test results compared to previous studies with more sensor data could support an efficiently trained ANN. However, a small training data set could result in the limited performance accuracy of the network, which was observed by an increase error in response to variations of the input. Since the GRF data are known to have high intra-subject variability, training with a larger dataset should produce improved prediction results for diverse test subjects.

Biomechanical knowledge about walking dynamics was used to design the ANN that predicts the unmeasured motion data from only a single IMU measurement. From the sacrum-mounted IMU data, the motion and force information of multiple joints were well predicted at various gait speeds by a simple ANN with a relatively small number of training datasets. This is attributed to the role of the CoM as a key dynamic descriptor of multi-segment lower limb coordination during the human walking behavior. The reliable prediction performance presented in this study implies the complementary roles of biomechanics domain knowledge and machine learning technology in predicting motion kinetics.

## Figures and Tables

**Table 1:** Speed of each subject’s slow, moderate, and fast speed used for data collection.

Subject	1	2	3	4	5	6	7	Average
Slow (m/s)	1.07	1.71	1.38	1.22	1.29	1.00	1.20	1.27 ± 0.23
Moderate (m/s)	1.21	1.87	1.49	1.37	1.51	1.14	1.36	1.42 ± 0.24
Fast (m/s)	1.54	2.26	1.78	1.76	2.07	1.49	1.76	1.81 ± 0.27

**Table 2:** Mean absolute error of heel-strike (HS) and toe-off (TO) timings with gait speed.

MAE (s)	Slow	Moderate	Fast	Total
HS	0.030 ± 0.022	0.025 ± 0.016	0.021 ± 0.017	0.025 ± 0.019
TO	0.016 ± 0.010	0.016 ± 0.011	0.010 ± 0.009	0.014 ± 0.010

**Table 3:** Mean absolute error and normalized root mean square error (NRMSE) of displacement and velocity of sacrum.

	Error	Slow	Moderate	Fast	Total
Vertical displacement	MAE (m)	0.006 ± 0.003	0.005 ± 0.002	0.006 ± 0.003	0.006 ± 0.003
	NRMSE (%)	20.12 ± 10.67	16.83 ± 7.14	16.82 ± 7.48	17.93 ± 8.71
A–P displacement	MAE (m)	0.06 ± 0.03	0.04 ± 0.02	0.04 ± 0.02	0.04 ± 0.03
	NRMSE (%)	8.76 ± 4.72	5.96 ± 3.75	4.69 ± 2.90	6.47 ± 4.21
Vertical velocity	MAE (m/s)	0.06 ± 0.02	0.06 ± 0.02	0.06 ± 0.03	0.06 ± 0.02
	NRMSE (%)	15.89 ± 5.82	14.21 ± 4.84	12.77 ± 4.43	14.29 ± 5.22
A–P velocity	MAE (m/s)	0.14 ± 0.079	0.12 ± 0.07	0.16 ± 0.08	0.14 ± 0.08
	NRMSE (%)	43.98 ± 24.94	34.87 ± 20.82	43.96 ± 23.40	40.94 ± 23.48

**Table 4:** Percentage of NRMSE values of all subjects as a function of the gait speed.

	Speed	Slow	Moderate	Fast
Segment angles of stance leg	Thigh	8.25 ± 1.40	7.70 ± 1.82	7.58 ± 2.66
	Shank	3.69 ± 1.30	3.66 ± 1.81	3.46 ± 0.66
	Foot	4.06 ± 1.07	4.24 ± 1.27	4.23 ± 0.89
Segment angles of swing leg	Thigh	9.42 ± 2.13	8.89 ± 2.52	7.94 ± 2.31
	Shank	5.82 ± 1.41	5.40 ± 1.13	4.99 ± 1.43
	Foot	6.43 ± 1.93	5.89 ± 1.20	5.64 ± 1.00
Joint torques of stance leg	Hip	11.67 ± 2.03	10.74 ± 1.26	10.65 ± 1.06
	Knee	10.58 ± 1.53	9.63 ± 1.40	9.33 ± 3.42
	Ankle	9.63 ± 3.12	9.24 ± 1.91	9.37 ± 1.68
Ground reaction forces	Vertical	6.80 ± 2.85	6.26 ± 1.24	8.21 ± 3.63
	A–P	6.49 ± 2.51	6.16 ± 1.76	6.70 ± 2.99

**Table 5:** Percentage of NRMSE values of specific subjects with different error levels.

	Error	Minimum	Median	Maximum
Segment angles of stance leg	Thigh	5.80 ± 0.93	7.45 ± 1.41	8.59 ± 1.11
	Shank	2.99 ± 0.80	2.90 ± 0.13	6.04 ± 1.55
	Foot	4.36 ± 0.78	3.76 ± 0.39	5.93 ± 1.35
Segment angles of swing leg	Thigh	6.43 ± 1.02	9.72 ± 1.23	12.33 ± 0.80
	Shank	4.09 ± 0.36	6.79 ± 0.99	6.32 ± 0.62
	Foot	5.33 ± 0.43	5.53 ± 0.20	7.73 ± 2.24
Joint torques of stance leg	Hip	11.95 ± 0.64	12.14 ± 1.40	10.91 ± 1.75
	Knee	8.32 ± 1.09	9.05 ± 0.87	9.72 ± 0.31
	Ankle	8.25 ± 0.12	7.78 ± 0.50	11.78 ± 0.25
Ground reaction forces	Vertical	4.63 ± 0.50	7.84 ± 0.65	6.03 ± 2.82
	A–P	5.49 ± 0.41	5.28 ± 0.54	4.91 ± 0.65

**Table 6:** Percentage of NRMSE values of different kinematics combinations.

Inputs	*x*, *t*	*x*, *v*, *t*	*v*, *t*	*x*, *v*, *a*, *t*	*v*, *a*, *t*
Segment angles of stance leg	5.20 ± 2.42	5.23 ± 2.69	7.62 ± 2.71	5.21 ± 2.38	6.34 ± 2.71
Segment angles of swing leg	7.22 ± 2.19	6.76 ± 2.30	8.44 ± 2.81	6.71 ± 2.25	8.37 ± 2.88
Joint torques of stance leg	10.04 ± 2.64	10.44 ± 2.63	12.25 ± 3.57	10.09 ± 2.10	11.56 ± 3.91
GRF	6.95 ± 2.58	6.76 ± 2.31	10.18 ± 3.17	6.77 ± 2.55	8.06 ± 2.34
Total	7.39 ± 3.04	7.35 ± 3.19	9.57 ± 3.57	7.24 ± 2.95	8.63 ± 3.63

**Table 7:** Percentage of NRMSE values of ANN trained with moderate speed only.

	Speed	Slow	Moderate	Fast
Joint torques of stance leg	Hip	13.41 ± 2.91	11.42 ± 0.65	11.07 ± 1.41
	Knee	11.35 ± 2.64	10.11 ± 2.23	9.92 ± 2.94
	Ankle	10.38 ± 3.94	9.30 ± 2.29	9.41 ± 1.95
Ground reaction forces	Vertical	6.64 ± 2.00	6.84 ± 1.30	7.93 ± 1.61
	A–P	6.24 ± 2.05	5.93 ± 0.85	7.09 ± 2.83

**Table 8:** Prediction errors of ground reaction forces (GRF) (previous studies and proposed method).

	S. E. Oh et al. (2013)		G. Leporace et al. (2018)		Proposed Method	
Number of subjects	48		17		7	
Measurement	11 Optical markers		2 IMUs at each shank		1 IMU at sacrum	
Prediction method	GRNN		FFNN		FFNN	
Prediction parameters	rRMSE (%)	*ρ*	MAD (%)	*ρ*	NRMSE (%)	*ρ*
Vertical GRF	5.8 ± 1.0	0.98	4.6 ± 0.7	0.97	6.26 ± 1.24	0.96 ± 0.03
A–P GRF	7.3 ± 0.8	0.97	4.0 ± 0.8	0.98	6.16 ± 1.76	0.98 ± 0.01
ML GRF	19.8 ± 2.2	0.92	10.5 ± 3.3	0.80	-	-

GRNN: General-regression neural network; FFNN: Feedforward neural network.

**Table 9:** Prediction errors of stance leg angle (previous studies and proposed method).

	A Findlow et al. (2008)		T.P. Luu et al. (2014)		Proposed Method	
Number of subjects	8		17		7	
Measurement	4 IMUs at each shank and feet		2 Gait parameters + 4 Anthropometric data		1 IMU at sacrum	
Prediction method	GRNN		GRNN		FFNN	
Prediction parameters (proposed)	MAD (degree)	*ρ*	MAD (degree)	*ρ*	RMSE (degree)	*ρ*
Hip (thigh)	8.64 ± 1.45	0.80 ± 0.05	3.73 ± 1.64	0.98 ± 0.03	3.14 ± 1.49	0.99 ± 0.03
Knee (shank)	7.14 ± 1.33	0.89 ± 0.05	5.41 ± 2.01	0.97 ± 0.04	2.17 ± 1.23	0.99 ± 0.00
Ankle (foot)	4.91 ± 0.76	0.75 ± 0.06	3.58 ± 1.44	0.92 ± 0.07	3.35 ± 1.58	0.99 ± 0.01

**Table 10:** Prediction errors of joint torques (previous studies and proposed method).

	M. M. Ardestani et al. (2014)		M. Mundt et al. (2018)		Proposed Method	
Number of subjects	4		12		7	
Measurement	14 sEMGs		3D joint angles		1 IMU at sacrum	
Prediction method	WNN		LSTM		FFNN	
Prediction parameters	rRMSE (%)	*ρ*	MAD (%)	*ρ*	NRMSE (%)	*ρ*
Hip	6.42	0.93	18.15	0.97	10.74 ± 1.26	0.90 ± 0.04
Knee	4.30	0.98	13.50	0.93	9.63 ± 1.40	0.96 ± 0.03
Ankle	4.20	0.98	6.41	0.98	9.24 ± 1.91	0.98 ± 0.01

WNN: Wavelet neural network; LSTM: Long-short term memory recurrent neural network.

**Figure A1:**
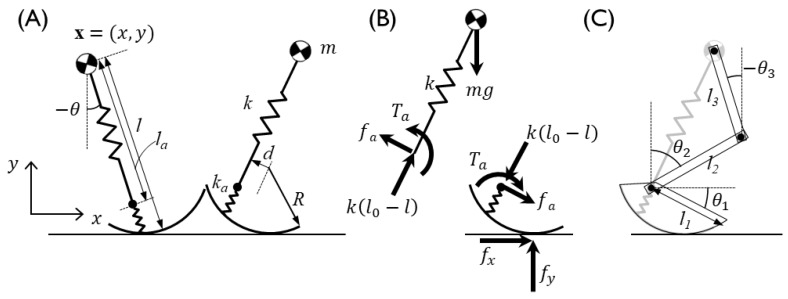
Compliant walking model with an off-centered, curvy foot combined with a springy foot-ankle segment [29]. (**A**) The model parameters and state variables of the model, and (**B**) the force and torque components. The position of the CoM in the sagittal plane; *x* and *y* are the positions of the horizontal and vertical axes, respectively, and the model state variables *l* and *θ* are the total spring length and angle, respectively, with a positive sign in the clockwise direction with respect to the vertical. The length and stiffness of the CoM–ankle are represented by *l_a_* and *k_a_*, respectively. The off-centered curvy foot parameters *d* and *R* are the offset of the ankle from the center of the foot and the radius of the curvy foot, respectively. The forces at the ground contact point are presented by *f_x_* and *f_y_* in the horizontal and vertical directions, respectively. The constraint force, *f_a_*, acts on the ankle in the vertical direction of the spring force, and the constraint ankle torque, *T_a_*, is generated to prevent foot rotation relative to the leg rotation, with a positive sign in the extension direction. With the kinematic constraint and the positions of the CoM and the ankle, (**C**) multibody segment angles are determined by inverse kinematics. Segment lengths and angles are represented by *l* and *θ*, respectively, with subscripts 1~3, and angles are positive in the clockwise direction.

## Appendix A

To examine whether the joint kinetics could be predicted through the ANN with CoM kinematics as the input, we tried to approximate the joint kinetics as a form of weighted sum of the CoM kinematics. Noting that the CoM dynamics are demonstrated by the spring mechanics, we started with the equations of motion of the recently published spring loaded inverted pendulum (SLIP) model [29]. The position of the CoM, x = (*x*, *y*), is expressed as model parameters of the SLIP, such as the leg angle *θ*, the leg length *l*, the radius of the curvy foot R, the offset of the ankle joint with respect to the foot *d*, and the ground contact position *c_hs_*, with the subscript *hs* denoting values at the onset of the stance phase (Figure A1), as follows:

(A1) $$\begin{array}{l} \left\{ \begin{array}{l} x=R(\theta -\theta_{hs})+(l-\sqrt{R^{2}-d^{2}})\sin\theta -d\cos\theta +c_{hs}\\ y=R+(l-\sqrt{R^{2}-d^{2}})\cos\theta +d\sin\theta \end{array}\right.\\ \mathrm{where}\;c_{hs}=x_{hs}-(l_{hs}-\sqrt{R^{2}-d^{2}})\sin\theta_{hs}+d\cos\theta_{hs} \end{array}$$

By applying the small angle approximation and binomial approximation and neglecting higher-order terms in Equation (A1), we can obtain the linearized mapping equation from the CoM position **x** to the augmented state **z** = [*l*, *θ, lθ*]*^Τ^*, which includes the state parameters, *l* and *θ* of the SLIP model, and its nonlinear term *lθ* as follows:

(A2) $$\begin{array}{l} z=W_{1}x+b_{1}\\ \mathrm{where}\;W_{1}=\left[\begin{array}{cc} 0 & 1\\ y_{hs}^{-1} & 0\\ 1 & 0 \end{array}\right],b_{1}=\left[\begin{array}{c} 0\\ \alpha y_{hs}^{-1}\\ \alpha \end{array}\right] \end{array}$$

where *α* = *Rθ*_hs_ + *d* − *c*_hs_.

The relationship between the augmented state **z** and the GRF and ankle torque are obtained from the CoM of the SLIP model as follows [29]:

(A3) $$\begin{array}{c} \left\{ \begin{array}{l} f_{x}=k(l_{0}-l)\sin\theta -f_{a}\cos\theta \\ f_{y}=k(l_{0}-l)\cos\theta +f_{a}\sin\theta \\ T_{a}=l_{a}f_{a} \end{array}\right.\\ \mathrm{where}\;f_{a}=\frac{k(l_{0}-l)\left(R\sin\theta +d\right)}{l-\sqrt{R^{2}-d^{2}}+R\cos\theta },\;l_{a}=c_{l}l_{0}-c_{k}\left(l_{0}-l\right) \end{array}$$

where the spring parameters *k* and *l*_0_ are the stiffness and the rest length of the spring, respectively, and the geometric parameters of *R* and *d* are the radius of the curvy foot and the offset of the ankle joint from the center of the curved foot, respectively. *l_a_* and *k_a_* are the length and stiffness of the CoM–ankle, as shown in Figure A1. The constants *c_l_* and *c_k_* are the ratio of the rest length of *l_a_* to the rest length of *l* and the ratio of the total leg stiffness *k* to the CoM–ankle stiffness *k_a_*, respectively (Figure A1). The ankle torque *T_a_* is expressed as *f_a_* and *l_a_*, which are the constraint force applied to the ankle joint and the distance from the ankle to the CoM, respectively. By applying a small angle approximation, a small offset *d* approximation compared to the foot length *R*, such as *d/R* << 1, and the small length deviation approximation of the leg length *l*, such as *l = l*_0_(1 *+ δ*), with *δ* being assumed to be small, then *f_a_* is approximated as follows:

(A4) $$\{f_{a}\approx k(l_{0}-l)\left(R\theta +d\right)l_{0}{}^{-1}\left(2-l\cdot l_{0}{}^{-1}\right)$$

and the above Equation (A3) can be rewritten as follows:

(A5) $$\left\{ \begin{array}{l} f_{x}=k(l_{0}-l)\theta -k(l_{0}-l)\left(R\theta +d\right)l_{0}{}^{-1}\left(2-l\cdot l_{0}{}^{-1}\right)\\ f_{y}=k(l_{0}-l)+k(l_{0}-l)\left(R\theta +d\right)\theta l_{0}{}^{-1}\left(2-l\cdot l_{0}{}^{-1}\right)\\ T_{a}=\left(\left(c_{l}-c_{k}\right)l_{0}+c_{k}l\right)k(l_{0}-l)\left(R\theta +d\right)l_{0}{}^{-1}\left(2-l\cdot l_{0}{}^{-1}\right) \end{array}\right.$$

If we ignore higher-order terms of small value, such as *θ^2^* ≈ 0, then the augmented kinetics **f** = [*f_x_*, *f_y_*, *T_a_*]*^T^* can finally be obtained as follows:

(A6) $$\left[\begin{array}{c} f_{x}\\ f_{y}\\ T_{a} \end{array}\right]=\left[\begin{array}{ccc} kdl_{0}{}^{-1} & k\left(l_{0}-R\right) & k\left(Rl_{0}{}^{-1}-1\right)\\ -k & kd & -kdl_{0}{}^{-1}\\ -kdc_{l} & kRl_{0}c_{l} & -kRc_{l} \end{array}\right]\left[\begin{array}{c} l\\ \theta \\ l\theta \end{array}\right]+\left[\begin{array}{c} -kd\\ kl_{0}\\ kdl_{0}c_{l} \end{array}\right]$$

(A7) $$\begin{array}{c} f=W_{2}z+b_{2}\\ \mathrm{where}\;W_{2}=\left[\begin{array}{ccc} kdl_{0}{}^{-1} & k\left(l_{0}-R\right) & k\left(Rl_{0}{}^{-1}-1\right)\\ -k & kd & -kdl_{0}{}^{-1}\\ -kdc_{l} & kRl_{0}c_{l} & -kRc_{l} \end{array}\right],\;b_{2}=\left[\begin{array}{c} -kd\\ kl_{0}\\ kdl_{0}c_{l} \end{array}\right] \end{array}$$

With the specified positions of the CoM and the ankle joint, the segment angles of the thigh and shank can be kinematically constrained as a function of the augmented spring states *θ* as follows [29]:

(A8) $$\left\{ \begin{array}{l} \theta_{1}=\theta -\tan\left(\left(1-c_{k}\right)\left(l_{0}-l\right)l_{1}{}^{-1}\right)\\ \sin\theta_{2}=0.5l_{a}\sin\theta \cdot l_{2}{}^{-1}\pm \cos\theta \sqrt{1-{\left(0.5l_{a}l_{2}{}^{-1}\right)}^{2}}\\ \sin\theta_{3}=\left(l_{a}\sin\theta -l_{2}\sin\theta_{2}\right)l_{2}{}^{-1} \end{array}\right.$$

where subscripts 1, 2, and 3 denote the foot, shank, and thigh, respectively, and *l*_1_ is the same as *l*_2_.

By applying small angle approximation and a small ankle length compared to the leg lengths, such as *l_a_* < 2*l*_1_, the segment angles **θ** = [*θ*_1_, *θ*_2_, *θ*_3_]*^T^* can be rewritten as follows:

(A9) $$\left[\begin{array}{c} \theta_{1}\\ \theta_{2}\\ \theta_{3} \end{array}\right]=\left[\begin{array}{ccc} \left(1-c_{k}\right)l_{1}{}^{-1} & 1 & 0\\ \mp 0.25c_{l}c_{k}l_{0}l_{2}{}^{-2} & 0.5\left(c_{l}-c_{k}\right)l_{0}l_{2}{}^{-1} & 0.5c_{k}l_{2}{}^{-1}\\ \pm 0.25c_{l}c_{k}l_{0}l_{2}{}^{-2} & 0.5\left(c_{l}-c_{k}\right)l_{0}l_{2}{}^{-1} & 0.5c_{k}l_{2}{}^{-1} \end{array}\right]\left[\begin{array}{c} l\\ \theta \\ l\theta \end{array}\right]+\left[\begin{array}{c} -\left(1-c_{k}\right)l_{0}l_{1}{}^{-1}\\ \pm \left(1-0.125c_{l}\left(c_{l}-2c_{k}\right)l_{0}{}^{2}l_{2}{}^{-2}\right)\\ \mp \left(1-0.125c_{l}\left(c_{l}-2c_{k}\right)l_{0}{}^{2}l_{2}{}^{-2}\right) \end{array}\right]$$

(A10) $$\begin{array}{l} \theta =W_{3}z+b_{3}\\ \mathrm{where}\;W_{3}=\left[\begin{array}{ccc} \left(1-c_{k}\right)l_{1}{}^{-1} & 1 & 0\\ \mp 0.25c_{l}c_{k}l_{0}l_{2}{}^{-2} & 0.5\left(c_{l}-c_{k}\right)l_{0}l_{2}{}^{-1} & 0.5c_{k}l_{2}{}^{-1}\\ \pm 0.25c_{l}c_{k}l_{0}l_{2}{}^{-2} & 0.5\left(c_{l}-c_{k}\right)l_{0}l_{2}{}^{-1} & 0.5c_{k}l_{2}{}^{-1} \end{array}\right],\\ b_{3}=\left[\begin{array}{c} -\left(1-c_{k}\right)l_{0}l_{1}{}^{-1}\\ \pm \left(1-0.125c_{l}\left(c_{l}-2c_{k}\right)l_{0}{}^{2}l_{2}{}^{-2}\right)\\ \mp \left(1-0.125c_{l}\left(c_{l}-2c_{k}\right)l_{0}{}^{2}l_{2}{}^{-2}\right) \end{array}\right] \end{array}$$

In summary, the kinematics and kinetics of the lower limb can be approximated as a weighted sum of augmented states **z** of the spring mechanics and the bias as follows:

(A11) $$\left[\begin{array}{c} f\\ \theta \end{array}\right]=\left[\begin{array}{c} W_{2}W_{1}\\ W_{3}W_{1} \end{array}\right]x+\left[\begin{array}{c} W_{1}b_{1}+b_{2}\\ W_{3}b_{1}+b_{3} \end{array}\right]$$

From the approximated relationship between the CoM position **x** and the joint kinematics and kinetics [**f^T^**, **θ^T^**], we tried to predict the 11 unmeasured joint kinetics during walking from a single IMU measurement near the CoM.

**Table A1:** Percentage of NRMSE values of each subjects.

Subject	1	2	3	4	5	6	7
**Segment angles of stance leg**							
**Slow**							
Thigh	8.81	9.38	8.34	5.27	8.58	9.32	8.03
Shank	3.17	4.06	3.01	2.12	3.25	3.98	6.24
Foot	3.49	3.84	3.48	4.03	3.02	4.24	6.31
**Moderate**							
Thigh	5.45	10.04	8.19	5.26	9.48	7.64	7.86
Shank	2.58	4.25	2.94	3.16	3.10	2.12	7.48
Foot	4.06	3.82	4.21	3.80	3.31	3.47	7.04
**Fast**							
Thigh	3.81	7.27	5.82	6.87	11.96	7.47	9.87
Shank	2.42	3.71	2.76	3.70	3.60	3.61	4.40
Foot	2.94	3.65	3.59	5.25	4.46	5.31	4.43
**Segment angles of swing leg**							
**Slow**							
Thigh	9.91	8.99	10.55	5.57	8.24	10.36	12.33
Shank	7.92	5.25	5.97	4.43	3.85	6.78	6.51
Foot	5.99	5.07	5.29	5.58	4.71	8.85	9.53
**Moderate**							
Thigh	6.41	10.39	10.31	6.17	8.38	7.48	13.13
Shank	5.39	5.05	6.50	4.12	6.00	3.90	6.83
Foot	5.02	5.41	5.63	4.83	5.97	5.93	8.43
**Fast**							
Thigh	4.21	8.00	8.31	7.55	9.53	6.45	11.53
Shank	4.15	4.94	7.89	3.72	4.64	3.99	5.63
Foot	4.23	5.20	5.65	5.56	7.46	6.16	5.22
**Joint torques of stance leg**							
**Slow**							
Hip	11.27	8.45	13.55	12.65	9.44	13.46	12.85
Knee	10.81	11.34	8.70	9.51	10.47	13.43	9.78
Ankle	8.58	8.90	7.65	8.33	6.44	15.62	11.88
**Moderate**							
Hip	11.67	8.28	12.12	11.39	11.00	10.30	10.44
Knee	11.48	9.44	8.41	8.10	11.43	8.59	10.00
Ankle	7.96	8.99	8.33	8.11	7.41	11.94	11.97
**Fast**							
Hip	11.67	9.79	10.75	11.80	11.59	9.52	9.44
Knee	7.67	8.28	10.04	7.36	16.50	6.06	9.39
Ankle	9.51	8.53	7.35	8.32	11.81	8.58	11.49
**Ground reaction forces**							
**Slow**							
Vertical	8.02	6.02	7.66	4.49	5.30	12.24	3.87
A–P	11.00	4.57	4.95	5.14	6.56	8.79	4.38
**Moderate**							
Vertical	7.65	6.63	7.29	4.22	6.82	6.21	5.01
A–P	8.95	4.92	4.98	5.38	8.41	5.78	4.70
**Fast**							
Vertical	6.10	6.75	8.55	5.19	15.77	5.91	9.21
A–P	5.22	5.80	5.90	5.93	13.43	4.95	5.64

**Table A2:** R^2^ values of each subjects.

Subject	1	2	3	4	5	6	7
**Angles of stance leg**							
**Slow**							
Thigh	0.92	0.89	0.93	0.97	0.92	0.91	0.94
Shank	0.98	0.97	0.98	0.99	0.98	0.97	0.91
Foot	0.96	0.96	0.96	0.95	0.98	0.93	0.88
**Moderate**							
Thigh	0.97	0.88	0.93	0.94	0.91	0.94	0.94
Shank	0.99	0.97	0.98	0.98	0.98	0.99	0.88
Foot	0.95	0.96	0.95	0.96	0.97	0.96	0.86
**Fast**							
Thigh	0.99	0.95	0.97	0.96	0.85	0.95	0.91
Shank	0.99	0.98	0.99	0.98	0.98	0.97	0.96
Foot	0.98	0.97	0.97	0.94	0.96	0.94	0.95
**Angles of swing leg**							
**Slow**							
Thigh	0.90	0.91	0.90	0.97	0.93	0.90	0.87
Shank	0.95	0.98	0.97	0.98	0.99	0.96	0.96
Foot	0.96	0.97	0.97	0.97	0.98	0.91	0.91
**Moderate**							
Thigh	0.96	0.88	0.89	0.97	0.93	0.95	0.85
Shank	0.98	0.98	0.96	0.99	0.97	0.99	0.96
Foot	0.97	0.97	0.97	0.98	0.97	0.96	0.93
**Fast**							
Thigh	0.98	0.94	0.93	0.95	0.89	0.96	0.87
Shank	0.99	0.98	0.95	0.99	0.98	0.99	0.97
Foot	0.98	0.98	0.97	0.97	0.95	0.97	0.98
**Joint torques of stance leg**							
**Slow**							
Hip	0.42	0.76	0.31	0.45	0.69	0.22	0.42
Knee	0.80	0.73	0.85	0.84	0.79	0.70	0.81
Ankle	0.91	0.90	0.93	0.91	0.96	0.71	0.78
**Moderate**							
Hip	0.41	0.75	0.37	0.56	0.56	0.54	0.58
Knee	0.78	0.72	0.86	0.88	0.71	0.87	0.75
Ankle	0.92	0.88	0.92	0.90	0.93	0.85	0.72
**Fast**							
Hip	0.50	0.72	0.67	0.56	0.68	0.68	0.71
Knee	0.91	0.87	0.82	0.92	0.48	0.94	0.81
Ankle	0.87	0.90	0.92	0.88	0.83	0.90	0.76
**Ground reaction forces**							
**Slow**							
Vertical	0.88	0.82	0.87	0.95	0.94	0.70	0.96
A–P	0.79	0.96	0.95	0.95	0.92	0.84	0.96
**Moderate**							
Vertical	0.87	0.90	0.88	0.95	0.90	0.82	0.93
A–P	0.86	0.95	0.96	0.95	0.87	0.94	0.96
**Fast**							
Vertical	0.91	0.91	0.83	0.93	0.49	0.91	0.82
A–P	0.95	0.94	0.94	0.93	0.68	0.96	0.93

## References

[1] Crossley K., Bennell K., Wrigley T., Oakes B.W. (1999). Ground reaction forces, bone characteristics, and tibial stress fracture in male runners. Med. Sci. Sports Exerc..

[2] Van der Worp H., Vrielink J.W., Bredeweg S.W. (2016). Do runners who suffer injuries have higher vertical ground reaction forces than those who remain injury-free? A systematic review and meta-analysis. Br. J. Sports Med..

[3] Zadpoor A.A., Nikooyan A.A. (2011). The relationship between lower-extremity stress fractures and the ground reaction force: A systematic review. Clin. Biomech..

[4] Messier S.P., Legault C., Schoenlank C.R., Newman J.J., Martin D.F., DeVita P. (2008). Risk factors and mechanisms of knee injury in runners. Med. Sci. Sports Exerc..

[5] Stefanyshyn D.J., Stergiou P., Lun V.M., Meeuwisse W.H., Worobets J.T. (2006). Knee angular impulse as a predictor of patellofemoral pain in runners. Am. J. Sports Med..

[6] Allen J.L., Kautz S.A., Neptune R.R. (2014). Forward propulsion asymmetry is indicative of changes in plantarflexor coordination during walking in individuals with post-stroke hemiparesis. Clin. Biomech..

[7] Bowden M.G., Balasubramanian C.K., Neptune R.R., Kautz S.A. (2006). Anterior-posterior ground reaction forces as a measure of paretic leg contribution in hemiparetic walking. Stroke.

[8] Turns L.J., Neptune R.R., Kautz S.A. (2007). Relationships between muscle activity and anteroposterior ground reaction forces in hemiparetic walking. Arch. Phys. Med. Rehabil..

[9] Karatsidis A., Bellusci G., Schepers H., de Zee M., Andersen M., Veltink P. (2017). Estimation of ground reaction forces and moments during gait using only inertial motion capture. Sensors.

[10] Oh S.E., Choi A., Mun J.H. (2013). Prediction of ground reaction forces during gait based on kinematics and a neural network model. J. Biomech..

[11] Leporace G., Batista L.A., Nadal J. (2018). Prediction of 3D ground reaction forces during gait based on accelerometer data. Res. Biomed. Eng..

[12] Ngoh K.J.-H., Gouwanda D., Gopalai A.A., Chong Y.Z. (2018). Estimation of vertical ground reaction force during running using neural network model and uniaxial accelerometer. J. Biomech..

[13] Guo Y., Storm F., Zhao Y., Billings S., Pavic A., Mazzà C., Guo L.-Z. (2017). A new proxy measurement algorithm with application to the estimation of vertical ground reaction forces using wearable sensors. Sensors.

[14] Johnson W.R., Mian A., Robinson M.A., Verheul J., Lloyd D.G., Alderson J. Multidimensional ground reaction forces predicted from a single sacrum-mounted accelerometer via deep learning. Proceedings of the ISB/ASB 2019.

[15] Shang Y., Wah B.W. (1996). Global optimization for neural network training. Computer.

[16] Sexton R.S., Alidaee B., Dorsey R.E., Johnson J.D. (1998). Global optimization for artificial neural networks: A tabu search application. Eur. J. Oper. Res..

[17] Parsopoulos K.E., Vrahatis M.N. (2004). On the computation of all global minimizers through particle swarm optimization. IEEE Trans. Evolut. Comput..

[18] Goodfellow R.C., Dimitrakopoulos R. (2016). Global optimization of open pit mining complexes with uncertainty. Appl. Soft Comput..

[19] White H. (1989). Learning in Artificial Neural Networks: A Statistical Perspective. Neural. Comput..

[20] Twomey J.M., Smith A.E. (1993). Nonparametric error estimation methods for evaluating and validating artificial neural network prediction models. Intelligent Engineering Systems Through Artificial Neural Networks.

[21] Zurada J.M., Malinowski A., Cloete I. Sensitivity Analysis for Minimization of Input Data Dimension for Feedforward Neural Network. Proceedings of the IEEE International Symposium on Circuits and Systems—ISCAS’94.

[22] Fernando D.A.K., Shamseldin A.Y. (2009). Investigation of Internal Functioning of the Radial-Basis-Function Neural Network River Flow Forecasting Models. J. Hydrol. Eng..

[23] Johnson W.R., Mian A., Donnelly C.J., Lloyd D., Alderson J. (2018). Predicting athlete ground reaction forces and moments from motion capture. Med. Biol. Eng. Comput..

[24] Geyer H., Seyfarth A., Blickhan R. (2006). Compliant leg behaviour explains basic dynamics of walking and running. Proc. Biol. Sci..

[25] Jung C.K., Park S. (2014). Compliant bipedal model with the center of pressure excursion associated with oscillatory behavior of the center of mass reproduces the human gait dynamics. J. Biomech..

[26] Kim S., Park S. (2011). Leg stiffness increases with speed to modulate gait frequency and propulsion energy. J. Biomech..

[27] Lee M., Kim S., Park S. (2014). Resonance-based oscillations could describe human gait mechanics under various loading conditions. J. Biomech..

[28] Whittington B.R., Thelen D.G. (2009). A simple mass-spring model with roller feet can induce the ground reactions observed in human walking. J. Biomech. Eng..

[29] Lim H., Park S. (2018). Kinematics of lower limbs during walking are emulated by springy walking model with a compliantly connected, off-centered curvy foot. J. Biomech..

[30] Lim H., Park S. (2019). A bipedal compliant walking model generates periodic gait cycles with realistic swing dynamics. J. Biomech..

[31] Drillis R., Contini R., Bluestein M. (1969). Body Segment Parameters.

[32] Paszke A., Gross S., Chintala S., Chanan G., Yang E., DeVito Z., Lin Z., Desmaison A., Antiga L., Lerer A. Automatic differentiation in pytorch. Proceedings of the the 31st Conference on Neural Information Processing Systems (NIPS 2017).

[33] Vathsangam H., Emken B.A., Schroeder E.T., Spruijt-Metz D., Sukhatme G.S. (2010). Energy Estimation of Treadmill Walking using On-body Accelerometers and Gyroscopes. IEEE Eng Med Bio.

[34] Findlow A., Goulermas J.Y., Nester C., Howard D., Kenney L.P.J. (2008). Predicting lower limb joint kinematics using wearable motion sensors. Gait Posture.

[35] Kuo A.D. (2002). Energetics of actively powered locomotion using the simplest walking model. J. Biomech. Eng..

[36] Donelan J.M., Kram R., Kuo A.D. (2002). Mechanical work for step-to-step transitions is a major determinant of the metabolic cost of human walking. J. Exp. Biol..

[37] Zhang J.J., Fiers P., Witte K.A., Jackson R.W., Poggensee K.L., Atkeson C.G., Collins S.H. (2017). Human-in-the-loop optimization of exoskeleton assistance during walking. Science.

[38] Yeom J., Park S. (2011). A gravitational impulse model predicts collision impulse and mechanical work during a step-to-step transition. J. Biomech..

[39] Moustakidis S.P., Theocharis J.B., Giakas G. (2008). Subject Recognition Based on Ground Reaction Force Measurements of Gait Signals. IEEE Trans. Syst. Man Cybern. Part B.

[40] Horst F., Lapuschkin S., Samek W., Muller K.R., Schollhorn W.I. (2019). Explaining the unique nature of individual gait patterns with deep learning. Sci. Rep. UK.

[41] Luu T.P., Low K.H., Qu X.D., Lim H.B., Hoon K.H. (2014). An individual-specific gait pattern prediction model based on generalized regression neural networks. Gait Posture.

[42] Mundt M., Koeppe A., Bamer F., Potthast W., Markert B. (2018). Prediction of Joint Kinetics Based on Joint Kinematics Using Artificial Neural Networks. ISBS Proc. Arch..

[43] Ardestani M.M., Zhang X., Wang L., Lian Q., Liu Y.X., He J.K., Li D.C., Jin Z.M. (2014). Human lower extremity joint moment prediction: A wavelet neural network approach. Expert. Syst. Appl..

[44] Chapman R.M., Torchia M.T., Bell J.E., Van Citters D.W. (2019). Assessing Shoulder Biomechanics of Healthy Elderly Individuals During Activities of Daily Living Using Inertial Measurement Units: High Maximum Elevation Is Achievable but Rarely Used. J. Biomech. Eng..

[45] Kesar T.M., Binder-Macleod S.A., Hicks G.E., Reisman D.S. (2011). Minimal detectable change for gait variables collected during treadmill walking in individuals post-stroke. Gait Posture.

[46] McGinnis R.S., Cain S.M., Davidson S.P., Vitali R.V., Perkins N.C., McLean S.G. (2016). Quantifying the effects of load carriage and fatigue under load on sacral kinematics during countermovement vertical jump with IMU-based method. Sports Eng..

[47] Wouda F.J., Giuberti M., Bellusci G., Maartens E., Reenalda J., Van Beijnum B.-J.F., Veltink P.H. (2018). Estimation of vertical ground reaction forces and sagittal knee kinematics during running using three inertial sensors. Front. Physiol..

[48] Thiel D.V., Shepherd J., Espinosa H.G., Kenny M., Fischer K., Worsey M., Matsuo A., Wada T. Predicting ground reaction forces in sprint running using a shank mounted inertial measurement unit. Proceedings of the 12th Conference of the International Sports Engineering Association.

[49] Stetter B.J., Ringhof S., Krafft F.C., Sell S., Stein T. (2019). Estimation of Knee Joint Forces in Sport Movements Using Wearable Sensors and Machine Learning. Sensors.

[50] Kulmala J.P., Avela J., Pasanen K., Parkkari J. (2013). Forefoot Strikers Exhibit Lower Running-Induced Knee Loading than Rearfoot Strikers. Med. Sci. Sports Exerc..

[51] Napier C., MacLean C., Maurer J., Taunton J., Hunt M. (2018). Kinetic risk factors of running-related injuries in female recreational runners. Scand. J. Med. Sci. Sports.

[52] Yang J.H., Suh S.W., Sung P.S., Park W.H. (2013). Asymmetrical gait in adolescents with idiopathic scoliosis. Eur. Spine J..

[53] Gonzalez I., Fontecha J., Hervas R., Bravo J. (2016). Estimation of Temporal Gait Events from a Single Accelerometer Through the Scale-Space Filtering Idea. J. Med. Syst..

[54] Storm F.A., Buckley C.J., Mazzà C. (2016). Gait event detection in laboratory and real life settings: Accuracy of ankle and waist sensor based methods. Gait Posture.

[55] Caldas R., Mundt M., Potthast W., Neto F.B.D., Markert B. (2017). A systematic review of gait analysis methods based on inertial sensors and adaptive algorithms. Gait Posture.

[56] McCamley J., Donati M., Grimpampi E., Mazza C. (2012). An enhanced estimate of initial contact and final contact instants of time using lower trunk inertial sensor data. Gait Posture.

[57] Forsman P.M., Toppila E.M., Haeggstrom E.O. Wavelet Analysis to Detect Gait Events. Proceedings of the 2009 Annual International Conference of the IEEE Engineering in Medicine and Biology Society.

